# Identification of 3’-UTR single nucleotide variants and prediction of select protein imbalance in mesial temporal lobe epilepsy patients

**DOI:** 10.1371/journal.pone.0252475

**Published:** 2021-06-04

**Authors:** Tanusree Chaudhuri, Janaki Chintalapati, Madhusoodan Vijayacharya Hosur

**Affiliations:** 1 Department of Natural Sciences and Engineering, National Institute of Advanced Studies, IISc campus, Bangalore, India; 2 CDAC-Centre for Development of Advanced Computing, Byappanahalli, Bangalore, India; University of Modena and Reggio Emilia, ITALY

## Abstract

The genetic influence in epilepsy, characterized by unprovoked and recurrent seizures, is through variants in genes critical to brain development and function. We have carried out variant calling in Mesial Temporal Lobe Epilepsy (MTLE) patients by mapping the RNA-Seq data available at SRA, NCBI, USA onto human genome assembly hg-19. We have identified 1,75,641 SNVs in patient samples. These SNVs are distributed over 14700 genes of which 655 are already known to be associated with epilepsy. Large number of variants occur in the 3’-UTR, which is one of the regions involved in the regulation of protein translation through binding of miRNAs and RNA-binding proteins (RBP). We have focused on studying the structure-function relationship of the 3’-UTR SNVs that are common to at-least 10 of the 35 patient samples. For the first time we find SNVs exclusively in the 3’-UTR of *FGF12*, *FAR1*, *NAPB*, *SLC1A3*, *SLC12A6*, *GRIN2A*, *CACNB4* and *FBXO28* genes. Structural modelling reveals that the variant 3’-UTR segments possess altered secondary and tertiary structures which could affect mRNA stability and binding of RBPs to form proper ribonucleoprotein (RNP) complexes. Secondly, these SNVs have either created or destroyed miRNA-binding sites, and molecular modeling reveals that, where binding sites are created, the additional miRNAs bind strongly to 3’-UTR of only variant mRNAs. These two factors affect protein production thereby creating an imbalance in the amounts of select proteins in the cell. We suggest that in the absence of missense and nonsense variants, protein-activity imbalances associated with MTLE patients can be caused through 3’-UTR variants in relevant genes by the mechanisms mentioned above. 3’-UTR SNV has already been identified as causative variant in the neurological disorder, Tourette syndrome. Inhibition of these miRNA-mRNA bindings could be a novel way of treating drug-resistant MTLE patients. We also suggest that joint occurrence of these SNVs could serve as markers for MTLE. We find, in the present study, SNV-mediated destruction of miRNA binding site in the 3’-UTR of the gene encoding glutamate receptor subunit, and, interestingly, overexpression of one of this receptor subunit is also associated with Febrile Seizures.

## Introduction

Epilepsy is amongst the most common neurological disorders, affecting up to 1% of the population of all ages [1]. Epilepsy is characterised by recurrent unprovoked seizures, and people with epilepsy have a 1.6- to 11.4-times greater mortality rate than the normal population. Though epilepsy can have both genetic and acquired causes, in about 60% of cases, the cause is not known [2–4]. The genetic factors are the variations that can occur in both coding and non-coding regions of the genes. The number of genes associated with epilepsy is continuously rising, and presently more than 900 genes are known [5,6]. The target gene luciferase assay (reporting gene technology), developed only recently, reveals that variants in the non-coding regions significantly influence epilepsy and other neurological disorders [7–10]. In a comparative study of 237 ion channel genes from neurologically normal individuals (n = 139) and idiopathic generalized epilepsy patients (n = 152) [11], 1.4% SNVs were found in the 3’-UTR regions. It is suggested that excess suppression of target mRNAs by miRNA binding in the 3’-UTR region disturbs the balance between neuronal excitation and neuronal inhibition thereby leading to epileptic seizures [12].

We report here identification of genomic variants through a comparative analysis of the RNA-seq data from 35 MTLE patient and three unrelated healthy individuals’ samples. We also report the structural and functional consequences of a chosen few variants in the 3’-UTR regions. We find the large number of SNVs (175641) distributed over 14700 genes of which 12333 are in the 655 genes already known, by earlier experimental data, to be related to epilepsy. The analysis identifies 33729 SNVs in the 3’-UTR region, and 2542 of these belong to 498 of the set of 655 epilepsy-related genes. Mechanisms of association with epilepsy of the remaining SNVs should be probed experimentally. SNVs in the 3’-UTR region have led to only creation of miRNA binding sites, and hence mRNA-translation repression, in the following genes: *FGF12*, *FAR1*, *NAPB*, *HECW2*, *NRG3*, *SLC1A3* and *SLC12A6*. On the other hand, loss of miRNA binding sites and hence de-repression of translation is predicted in the following genes: *MMADHC*, *FBXO28*, *GRIN2A*, *CACNB4*, *TDP2*, *ABHD12*, *RBPJ* and *PCMT1*. Molecular modelling reveals that the SNV has significantly affected the three-dimensional structure of the 71-residue 3’-UTR segment thereby disturbing formation of proper translation complex. Molecular modelling also reveals that the binding of miRNAs to 3’-UTR are strengthened by the SNV leading to greater translation repression. These results suggest that, even in the absence of missense variants that affect functionality of the protein, 3’-UTR SNVs can disturb protein balance leading to development of epileptic conditions [13]. Our study also points to the general possibility that combinations of independent variants in regulatory regions of different genes can be markers for epilepsy. We find a probable linkage between Febrile seizures and MTLE through genetic signals for over-expression of glutamate receptor subunits in both.

## Results

The SRA Ids of the RNA-Seq data and statistics of mapping to hg-19 are given in S1 Table.

**Fig 1:**
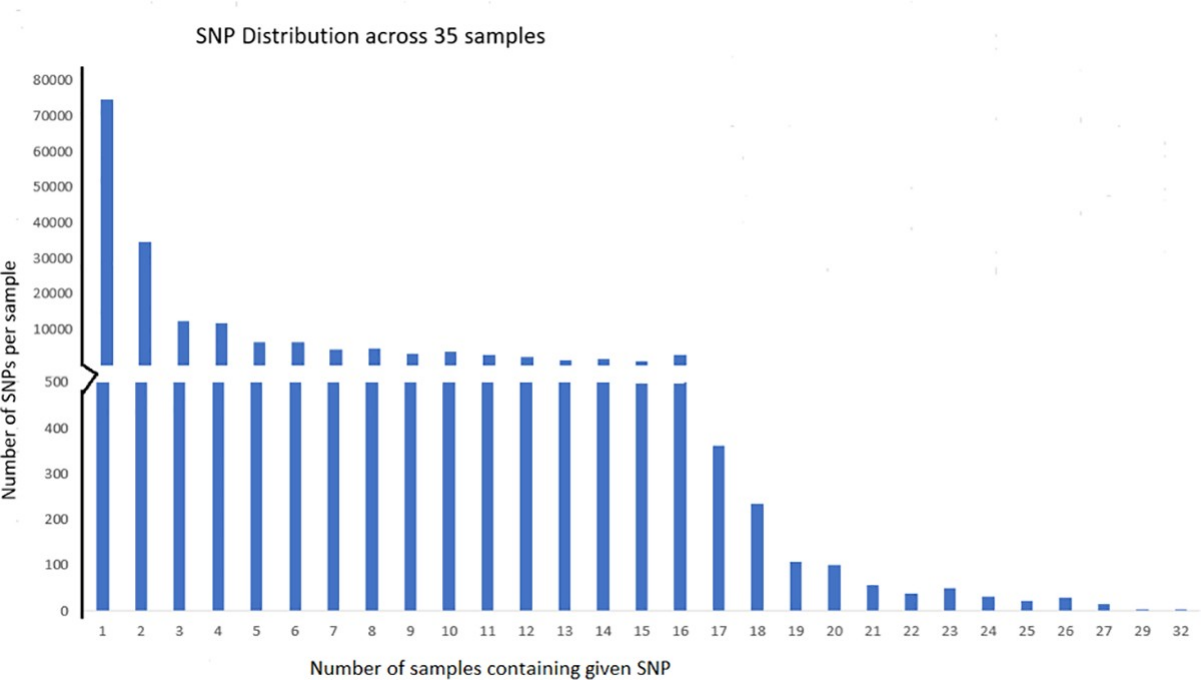
SNV distribution across 35 samples.

As may be seen, more than 90% of paired reads from each sample are mapped onto hg-19. Distribution of the variants common to mapping separately by Bowtie2 and BWE is shown in Fig 1 as a function of number of samples in which a given variant is occurring.

There is a total of 1,75,641 SNVs occurring in 14700 genes when a DP (depth of read) cut-off of 100 is used to accept a variant as genuine. Of these, 12333 SNVs are present in the 655 genes that are already known to be associated with epilepsy. The relevance of remaining SNVs to epilepsy should be explored experimentally. The distribution of variants (SNVs) into five categories of missense variants, 3’-UTR variants, 5’-UTR variants, intronic variants and other variants, is given in Table 1. The category ’other’ contains downstream as well as synonymous and remaining variant types, depending on their position on the genome. There are 210 missense variants distributed over 121 epilepsy-associated genes, while the corresponding numbers for 3’-UTR variants are 2542 and 498 respectively (Table 1). The names of the genes carrying these regulatory 5’-UTR SNVs and 3’-UTR SNVs are listed in S2 Table.

**Table 1:** Distribution of SNV’s into different consequences with a DP cutoff of 100.

Sr. No.	Consequence	Number of alterations (SNV and INDEL) Dpmin = 100	Number of SNVs in epilepsy-related genes (Dpmin = 100)	Number of epilepsy-associated genes with SNVs
1	Missense variants	2481/175641	210/12333	121/655
2	3’-UTR	33729/175641	2542/12333	498/655
3	5’-UTR	1158/175641	77/12333	52/655
4	Intronic variants	50946/175641	5343/12333	296/655
5	Others	87327 /175641	4161 /12333	579/655

In S2 Table we have highlighted those genes which are targets of miRNAs that are found to be overexpressed in MTLE patients with Hippocampal Sclerosis (HS) [14]. From among the total 3’-UTR SNVs, we have selected for further analysis those SNVs which are present in ten or more of the 35 patient samples. We find that SNVs in the 3’-UTR regions have created miRNA binding sites in 18 genes and destroyed them in 19 genes, and these genes are listed in Table 2. The total number of binding sites created is 51 while the total number of binding sites lost is 69 (Table 2). Information about the degree of relatedness to epilepsy of each affected gene, as classified by Wang et al [13], is also given in Table 2. For some of the genes, there is both a loss and gain in miRNA binding sites (Table 2), and these genes have not been analysed further here.

**Table 2:** Numbers of miRNA binding sites, gained and lost, due to observed SNVs in the 3’-UTR regions of the epilepsy-associated genes.

Sr. No.	Gene name (relatedness to epilepsy)	Number gained, (miRNAs)	Number lost, (miRNAs)	Biological function
1	*FGF12* (EG^$^)	1 (hsa-miR-4760-3p)	Nil	Involved in the positive regulation of voltage-gated sodium channel activity in Epilepsy.
2	*ATP1A2* (ERG)	11 (hsa-miR-4271, hsa-miR-4725-3p, hsa-miR-4747-5p, hsa-miR-6780b-5p, hsa-miR-6783-5p, hsa-miR-3161, hsa-miR-371a-3p, hsa-miR-152-5p, hsa-miR-204-3p, hsa-miR-4314, hsa-miR-4646-5p)	14 (hsa-miR-1224-3p, hsa-miR-1260a, hsa-miR-1260b, hsa-miR-3153, hsa-miR-4270, hsa-miR-4502, hsa-miR-4713-5p, hsa-miR-532-3p, hsa-miR-5584-5p, hsa-miR-6733-3p, hsa-miR-6733-5p, hsa-miR-6739-5p, hsa-miR-6750-5p)	Abnormal Na+/K+ ATPase system function disrupts the K+ gradient and impairs glutamate clearance, which likely contributes to epilepsy [15–17].
3	*SLC1A3* (ERG)	2 (hsa-miR-3668, hsa-miR-576-3p)	Nil	Glutamate transport & ion-flux.
4	*TUBB4A* (ERG)	1 (hsa-miR-4742-5p)	5 (hsa-miR-4270, hsa-miR-4763-3p, hsa-miR-6722-3p, hsa-miR-6754-5p, hsa-miR-9500)	Microtubule subunit.
5	*RFX3* (Potential EAG)	4 (hsa-miR-552-5p, hsa-miR-4674, hsa-miR-760, hsa-miR-7158-5p)	8 (hsa-miR-1291, hsa-miR-146b-3p, hsa-miR-339-5p, hsa-miR-4421, hsa-miR-5699-3p, hsa-miR-6724-5p, hsa-miR-6773-5p, hsa-miR-6775-3p)	Regulatory Factor X3, acts as a transcription factor.
6	*FAR1* (ERG)	4 (hsa-miR-497-3p, hsa-miR-548aa, hsa-miR-548ap-3p, hsa-miR-548t-3p)	Nil	Lipid synthesis, variants and FAR1 deficiency [18] is associated with early-onset epilepsy.
7	*GRIN2B* (EG)	3 (hsa-miR-124-5p,hsa-miR-498-5p,hsa-miR-513b-3p)	1 (hsa-miR-3974)	The GluN2B subunit of N-methyl-d-aspartate receptors responsible for Ca2+ permeability in excitatory synaptic transmission of CNS [19].
8	*NAPB* (Potential EAG)	3 (hsa-miR-6818-5p, hsa-miR-6867-5p, hsa-miR-3177-5p)	Nil	Metal ion binding
9	*CNTNAP2* (Neurodevelopment associated EG)	1 (hsa-miR-4766-5p)	2 (hsa-miR-1299, hsa-miR-875-3p)	CNTNAP2 encodes Caspr2. Targeted disruption of which results in reduction in the accumulation of K+ channels [20].
10	*HECW2* (Potential EAG)	1 (hsa-miR-136-5p)	Nil	Ligase. Variants associated with epilepsy. Low levels associated with Hirschsprung’s disease, which is constipation in children.
11	*NRG3* (Potential EAG)	1 (hsa-miR-580-3p)	Nil	Signal receptor binding. Schizophrenia.
12	*ACADSB* (Not found)	4 (hsa-miR-4499, hsa-miR-548u, hsa-miR-7161-5p, hsa-miR-4724-3p)	2 (hsa-miR-1257, hsa-miR-5586-3p)	ACADSB is associated with autosomal recessive SBCAD deficiency.
13	*GALC* (ERG)	1 (hsa-miR-556-3p)	5 (hsa-miR-3158-5p, hsa-miR-4418, hsa-miR-509-3-5p, hsa-miR-509-5p, hsa-miR-574-3p)	Hydrolysis galactose.
14	*SLC12A6* (Neurodevelopment associated EG)	4 (hsa-miR-143-3p, hsa-miR-4756-3p, hsa-miR-4770, hsa-miR-6088)		K-Cl co-transporter. Neuropathy. Mediates electroneutral potassium-chloride co-transport.
15	*ATP8A2* (Potential EAG)	2 (hsa-miR-186-3p, hsa-miR-4422)	2 (hsa-miR-570-3p, hsa-miR-645)	Helps in maintaining asymmetry in membrane lipids.
16	*HERC2* (ERG)	6 (hsa-miR-30c-1-3p, hsa-miR-30c-2-3p, hsa-miR-5192, hsa-miR-6731-5p, hsa-miR-6788-5p, hsa-miR-8085)	9 (hsa-miR-1273h-5p, hsa-miR-149-3p, hsa-miR-30b-3p, hsa-miR-6779-5p, hsa-miR-6785-5p, hsa-miR-6795-5p, hsa-miR-6805-5p, hsa-miR-6883-5p, hsa-miR-6887-5p)	Dysregulation of HERC2 and HERC1 proteins is associated with severe human diseases such as neurological disorders and cancer [21].
17	*MAPRE2* (ERG)	1 (hsa-miR-4255)	3 (hsa-miR-4738-3p, hsa-miR-548g-3p, hsa-miR-892c-3p)	MAPRE2 encodes a microtubule-associated protein, which is a central regulator of microtubule dynamics and reorganization during cell differentiation [22]
18	*PGAP1* (ERG)	1 (hsa-miR-510-3p)	4 (hsa-miR-1250-3p, hsa-miR-3617-5p, hsa-miR-4635, hsa-miR-641)	Defect of PGAP1 leads to GPI-anchors with an abnormal structure and altered biochemical properties [23].
19	*MMADHC* (ERG)	Nil	1 (hsa-miR-1277-5p)	Vitamin B12 metabolissm.
20	*FBXO28* (Potential EAG)	Nil	4 (hsa-miR-135a-3p, hsa-miR-3162-5p, hsa-miR-3163, hsa-miR-5700)	Ubiquitination and degradation. Chromosome deletion syndrome.
21	*GRIN2A* (EG)	Nil	3 (hsa-miR-208a-5p, hsa-miR-208b-5p, hsa-miR-627-3p)	NMDA receptor subunit. Epilepsy.
22	*CACNB4* (EG)		1 (hsa-miR-3165)	Calcium channel protein. Generalised epilepsy
23	*TDP2* (ERG)	Nil	1 (hsa-miR-607)	Phosphodiesterase. Single strand DNA binding. Ataxia.
24	*ABHD12* (ERG)	Nil	1 (hsa-miR-4485-5p)	Hydrolytic enzyme. Polyneuropathy, Ataxia.
25	*RBPJ* (Potential EAG)	Nil	1 (hsa-miR-4666a-5p)	DNA-binding TF.
26	*PCMT1* (Not found)	Nil	1 (hsa-miR-584-5p)	Methyl transferase. Diabetes.

$Note: EG = Epilepsy Genes, ERG = Epilepsy Related Genes [24].

Only SNVs present in at least 10 samples are considered.

**Fig 2:**
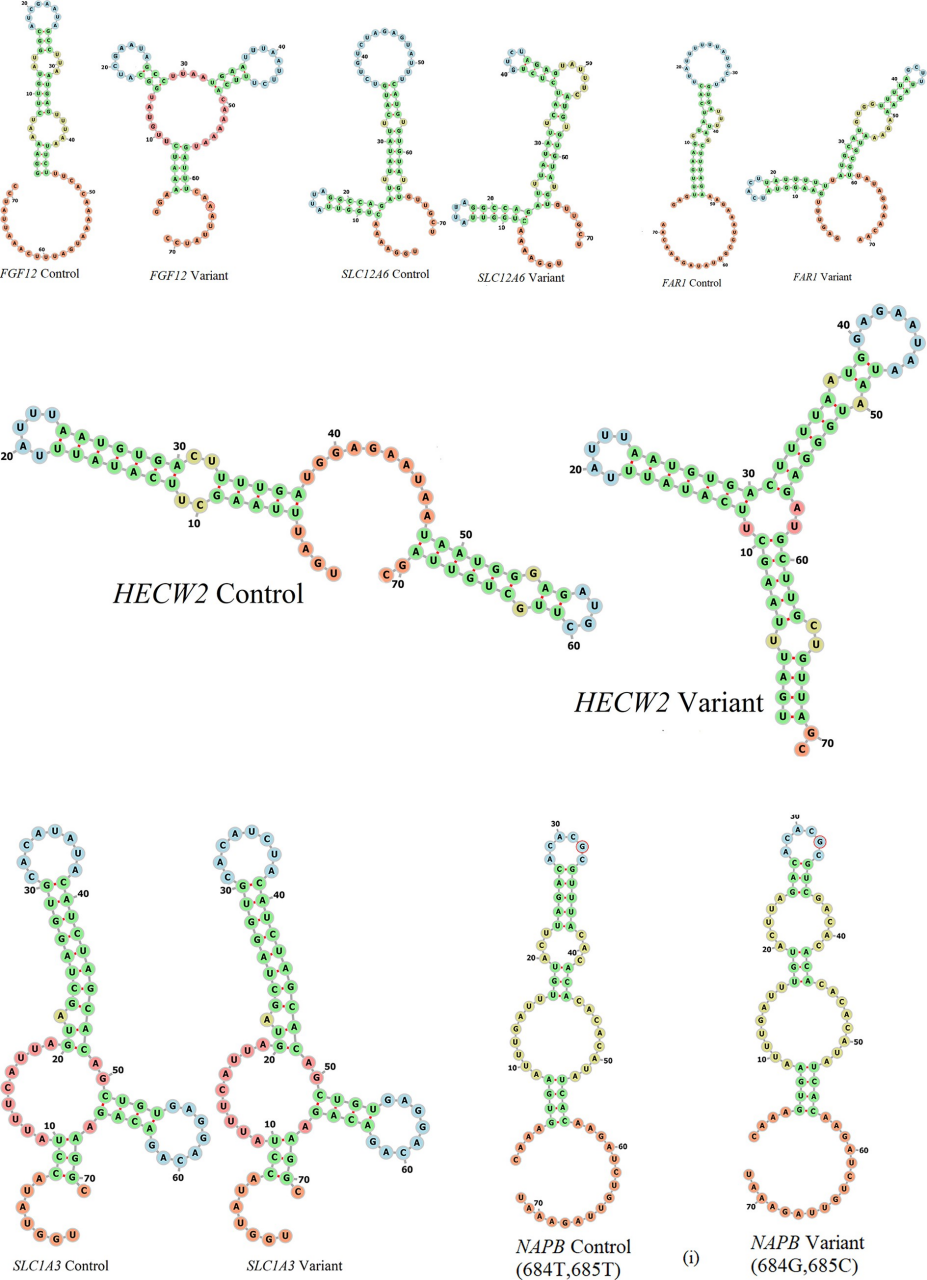
a: Secondary structure changes in FGF12 due to G to A SNV. b: Secondary structure changes in SLC12A6 due to G to C SNV. c: Secondary structure changes in FAR1 due to A to G SNV. d: Secondary structure changes in HECW2 due to C>T SNV. e: Secondary structure changes in SLC1A3 due to A>C SNV. f: NAPB (i): SNV positions 684 & 685 U>C and U>G; (ii): SNV position at 1151 is C>U).

The following genes have only gained in miRNA binding sites due to the SNV: *FGF12*, *FAR1*, *NAPB*, *SLC1A3*, *HECW2*, and *SLC12A6*. Figure pairs in Fig 2A–2F show comparisons of predicted secondary structures for the native (left) and variant (right) mRNA segment of length 71 residues containing the SNV, for each of these genes. In all mRNA segments chosen, the SNV is located at position 36 in the sequence.

In *FGF12*, the missense variant R to H in its gene product, is the cause for early onset epilepsy [25,26]. In the present analysis of MTLE patients, this missense variant is not present, but there is a 3’-UTR SNV, G>A, instead (Fig 2A). It is very clear that the positions of loops and stems in the two are very different, and very likely this substantially alters the tertiary structure of this mRNA segment.

In *SLC12A6* [27,28] gene, the 3’-UTR SNV is a change from G to C (Fig 2B). There is an extra stem in the case of variant mRNA.

In *FAR1* [29] gene, the SNV is a change from residue A to G (Fig 2C). While A is part of a stem the G residue in the variant is part of a loop. There is also a significant change in the positions of loops and stems in the predicted secondary structures.

In *HECW2* the base change from C to T (Fig 2D) has generated an additional stem loop structure.

In *SLC1A3*, there is no difference in the secondary structure due to base changes from A to C (Fig 2E), but it has created binding sites for two additional miRNAs.

In *NAPB*, which is involved in the biological process of the vesicular release of glutamate from a pre-synapse, l [30], there are two positions with SNVs; one doublet at location 684–685 and another at location 1151. The SNVs at positions 684 & 685 are U>C and U>G (Fig 2F). The one at 1151 is C>U (Fig 2F).

The genes which display only loss of miRNA binding sites due to SNV are: *GRIN2A*, *CACNB4*, *FBXO28*, *TDP2*, *ABHD12*, *RBPJ*, *PCMT1*, and secondary structures for these are given in S1 Fig.

It is clear from these figures that there are significant differences in the secondary structures of the 3’-UTR segments with and without the SNV.

### Three dimensional structures of 3’-UTR segments

Three-dimensional structures for the 71 residue mRNA fragments for which secondary structures were presented above were built, and Table 3 gives the folding energies for the native and variant mRNA fragments. There is no definite correlation between folding energy and SNV that either creates or destroys miRNA binding site.

**Table 3:** Folding energy of the 71 residue 3’-UTR segments with and without the SNV.

Gene name	71 residue mRNA segment folding Energy, kcal/mole	
	Control	Variant
*FAR1*	-21.912	-38.692
*FGF12*	-24.769	-18.895
*GRINA*	-5.122	-11.936
*NAPB*	-53.706	-42.955
*SLC12A6*	-37.457	-22.047
*CACNB4*	-49.599	-45.455
*SLC1A3*	-31.985	-36.485
*FBXO28*	-6.727	-5.747
*HECW2*	-31.609	-39.255
*NRG3*	-21.065	-19.074
*MMADHC*	-25.422	-25.394
*TDP2*	-38.422	-38.485
*ABHD12*	-66.736	-66.806
*RBPJ*	-42.494	-49.846
*PCMT1*	-10.708	-19.128

**Fig 3:**
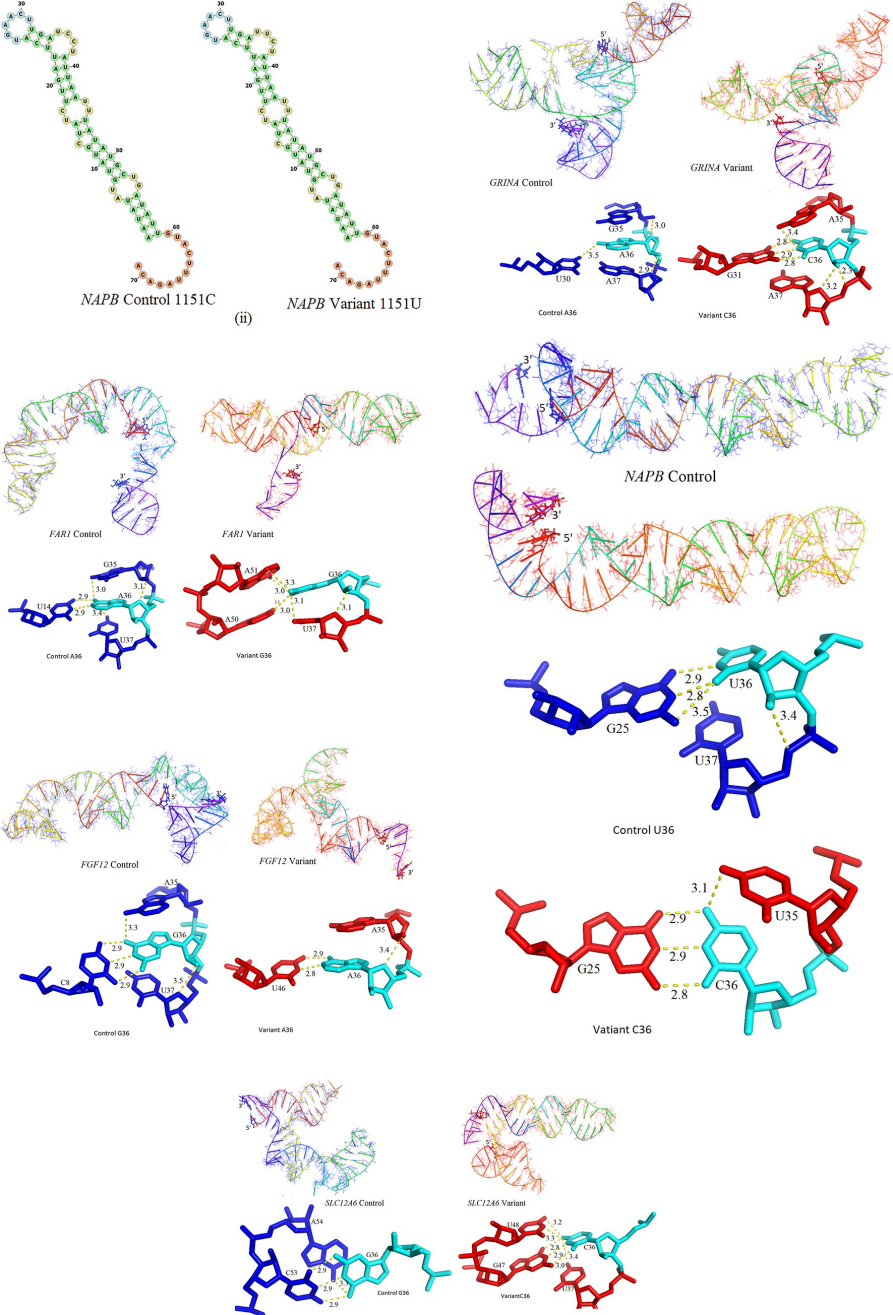
a: Structural comparisons (control: Blue, variant: Red). top) Structures of 3’-UTR segments of FAR1 control and variant. Only nucleotide bases are shown in stick representation. The structures and intra-molecular hydrogen bonds are radically different. bottom) hydrogen bonding patterns involving SNV base: left, A36 (control) and right, G36 (variant). b: Structural comparisons (control: blue, variant: red). top) Structures of 3’-UTR segments of FGF12 control and variant. Only nucleotide bases are shown in stick representation. The structures and intra-molecular hydrogen bonds are radically different. bottom) hydrogen bonding patterns involving SNV base: left, G36 (control) and right, A36 (variant). c: Structural comparisons (control: blue, variant: red). top) Structures of 3’-UTR segments of GRIN2A control and variant. Only nucleotide bases are shown in stick representation. The structures and intra-molecular hydrogen bonds are radically different. bottom) hydrogen bonding patterns involving SNV base: left, A36 (control) and right, C36 (variant). d: Structural comparisons (control: blue, variant: red). top) Structures of 3’-UTR segments of NAPB control and variant. Only nucleotide bases are shown in stick representation. The structures and intra-molecular hydrogen bonds are radically different. bottom) hydrogen bonding patterns involving SNV base: left, U36 (control) and right, C36 (Variant). e: Structural comparisons (control: blue, variant: red). top) Structures of 3’-UTR segments of SLC12A6 control and variant. Only nucleotide bases are shown in stick representation. The structures and intra-molecular hydrogen bonds are radically different. bottom) hydrogen bonding patterns involving SNV base: left, G36 (control) and right, C36 (variant). f: Structural comparisons (control: blue, variant: red). top) Structures of 3’-UTR segments of CACNB4 control and variant. Only nucleotide bases are shown in stick representation. The structures and intra-molecular hydrogen bonds are radically different. bottom) hydrogen bonding patterns involving SNV base: left, C36 (control) and right, U36 (variant).

For example, though miRNA binding sites are destroyed by the SNVs in both *GRIN2A* and *CACNB4*, the SNV in *GRIN2A* has stabilized the mRNA fragment while the SNV in *CACNB4* has destabilized the mRNA fragment. Similarly, though miRNA binding sites are created by the SNVs in both *SLC12A6* and *SLC1A3*, the SNV in *SLC1A3* has stabilized the mRNA fragment while the SNV in *SLC12A6* has destabilized the mRNA fragment. Three dimensional structures of the native/control as well as variant mRNA segments of few representative genes are shown in Fig 3A–3F as wire models. The hydrogen bonding interactions of the nucleotide base at position 36 (the SNV position) is also shown in Fig 3A–3F. The view direction for the pair of figures is identical. It is obvious that there are significant conformational differences brought about by the differences in the hydrogen bonding interactions involving the base at the SNV position. Similar figures for other genes are provided as S2 Fig.

Folding energy of the mRNA fragment is significant as it determines the affinity of binding in the bimolecular mRNA–miRNA complex.

### MicroRNA binding to 3’-UTR

MicroRNA binding to the target mRNAs is mediated through the argonaute proteins AGO1-4 [31]. The nucleotide residues at the 5’- and 3’- termini of the mature miRNA anchor the miRNA to the argonaut, and the seed region (residues 2–9) recognizes the target mRNA through Watson-Crick hydrogen bonding. The efficiency of binding depends on the accessibility of the mRNA and miRNA bases for canonical pairing, with higher folding energy making access more improbable. Different software packages have been developed after considerations of access, sequence conservation, degree of seed pairing and other factors to predict miRNAs that can bind at a given site. We have used software RIblast and the database miRSNPDB to search for mature miRNAs that can bind at the 3’-UTR SNV site. The software RNAcofold predicts the secondary structure, and using this as input, the software Rosetta builds the three-dimensional structure of the mRNA-miRNA complex and estimates the binding energies, and these estimates are given in Table 4.

**Table 4:** Binding energies for binding of miRNAs at sites created by 3’-UTR SNVs.

Gene Name	Name of miRNA binding at SNV	Binding Energy (kcal/mol)	Number of Seed region residues in base-pairing.
*FAR1*	hsa-miR-548aa	-13.0	8
	hsa-miR-548ap-3p	-11.3	8
	hsa-miR-548t-3p	-13.0	8
*FGF12*	hsa-miR-4760-3p	-6.0	7
*SLC12A6*	hsa-miR-143-3p	-9.2	7
	hsa-miR-4756-3p	-12.7	9
	hsa-miR-4770	-9.9	11
	hsa-miR-6088	-11.2	8
*NAPB*(1151C>T)	hsa-miR-3177	-12.7	12
*HERCW2*	hsa-miR-136-5p	-8.90	7
*NRG3*	hsa-miR-580-3p	-17.80	12
*SLC1A3*	hsa-miR-3668	-9.80	7
	hsa-miR-576-3p	-12.10	9

**Fig 4:**
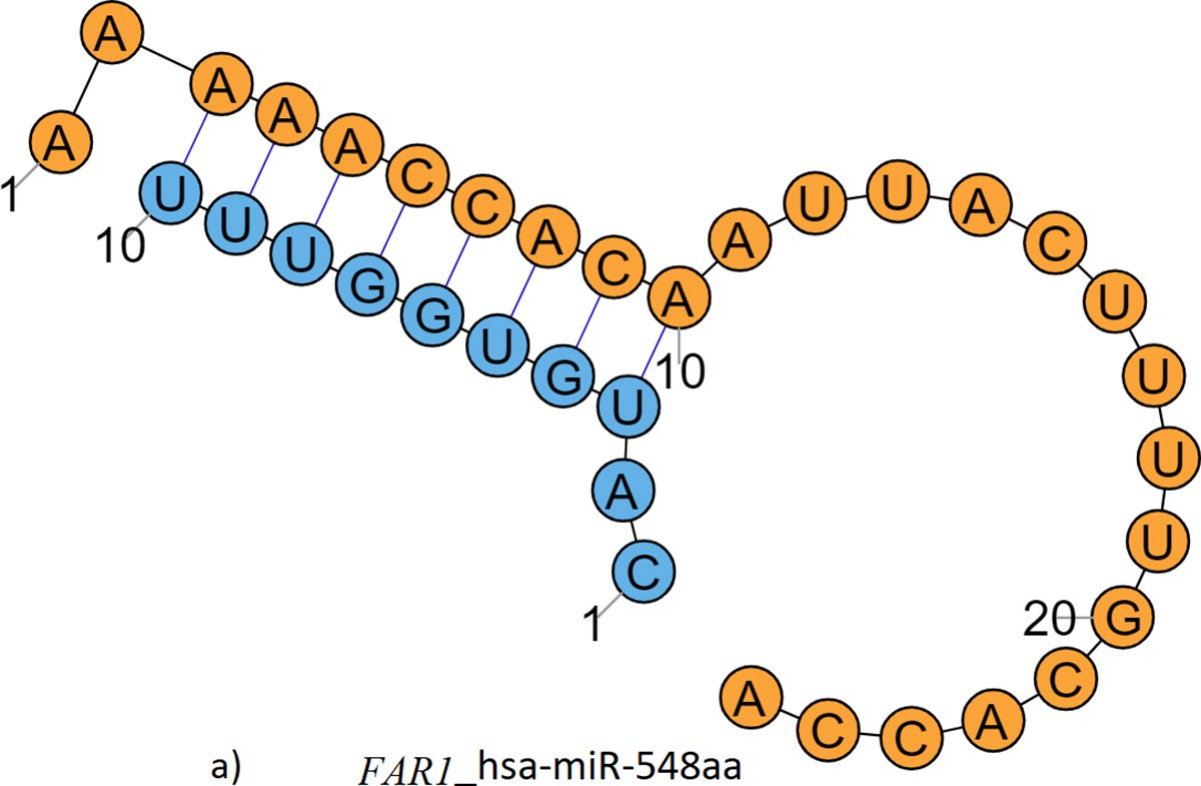
Schematic of mRNA-miRNA seed region hydrogen bond interactions in FGF12. (mRNA: Blue, miRNA: Scarlet).

For genes *FAR1*, *FGF12*, *NAPB*, *HERCW2*, *NRG3*, *SLC1A3* and *SLC12A6*, there are only creations of binding sites for miRNAs. Three additional miRNAs will bind to *FAR1* 3’-UTR, one each to *FGF12* and *NAPB*, and four in the case of *SLC12A6*. The number of Watson-Crick hydrogen bonded base-pairs in the miRNA-mRNA complex (including the seed region) is listed in the last column of Table 4. Hydrogen bonding interactions in the seed region of the modeled complex for *FGF12* is shown schematically in Fig 4. Similar figures for other genes are given in S3 Fig.

## Discussion

### Variant calling

Due to the drop in cost of sequencing, RNA-sequencing (RNA-seq) has become a powerful tool not only in research but also for clinical applications. Several workflows developed to identify variants by analysing RNA-seq data are found to perform equally well [32]. BWA and bowtie2 are splice-unaware short-read aligners that map nucleotide sequence data to a reference genome by minimising edit distances. We have used as reference genome the human genome assembly hg-19, which is not as complete and accurate as the latest assembly, GRCh38 [33]. Short reads may map equally well to multiple positions especially because nearly 50% of the human genome consists of repetitive elements. Additionally, sequencing errors may also lead to erroneous mapping of the short reads. These factors and the small sample size are some of the limitations of present study. Nevertheless, filtering of bases of low-quality mapping and the high depth of read we have prescribed before accepting a variant as genuine, would have reduced false positive SNVs. Detection of variants using RNA-seq data has one more limitation coming from the fact that the RNA-seq data depend on the degree of expression and stability of the mRNA. Further, variant alleles with low frequency values are poorly detected by the RNA-seq method [34]. Though final confirmation of variant-effect predictions have to come from experiments, computational methods suggest what experiments to do. It is encouraging that 3’-UTR SNVs in eight genes we have identified (Table 2) are mentioned in ClinVar database as being associated with epilepsy.

### Protein translation—Changes through miRNA binding sites

Twin studies and drug-response studies show that there is a complex genetic influence in epilepsy, the complexity stemming from the fact that epilepsy is very heterogeneous. Genetic influence is through variants causing dys-functioning in the expression and translation of a variety of genes [35,36]. The mechanisms are however not fully understood. Majority of earlier studies have concentrated on missense variants, and have shown that these variants cause biological effects such as decreased current amplitude, reduction in surface expression of functional receptors, alterations in the speeds of activation and deactivation of ion channels [26,27,29,30,37–39]. These effects originate from a paucity of properly functioning protein molecules at the desired locations within the cell. Studies using reporter gene assay have revealed, only recently, the importance of variants in non-coding 3’-UTR regions to several neurological disorders [40–43]. We have, therefore, chosen to identify and investigate structural consequences of the 3’-UTR SNVs occurring in at-least 10 of the 35 MTLE patients. These SNVs have the potential to create or destroy miRNA binding sites, thereby playing important roles in epilepsy since a number of miRNAs are differentially expressed in epilepsy patients [44]. As may be seen in Table 2, there is only creation of binding sites in *FGF12*, *FAR1*, *NAPB*, *SLC1A3* and *SLC12A6*. Interestingly, all these genes are already known to be associated with epilepsy because of missense variants in them [45–48]. For three of these genes, the 3’-UTR SNVs have created binding sites for more than one miRNA. The binding energies listed in Table 3 suggest strong binding of miRNAs at the SNV sites. Since each miRNA binding causes translational repression, the creation of miRNA binding sites will substantially reduce the availability of functional molecules, a feature also resulting from missense variants. We also found that in *GRIN2A*, *CACNB4* and *FBXO28*, 3’-UTR SNVs occur causing only loss of miRNA binding sites suggesting overproduction of these proteins. Our results, thus, predict shortage and over-abundance of selected proteins in MTLE patients. A significant reduction in the levels of SCN4B protein in the drug-resistant TLE patients compared to non-epileptic control specimens, has been demonstrated recently [49]. On the other hand, significant increase in levels of DNA methyl transferase enzymes has been associated with TLE [50].

### Protein translation—Repression by altered mRNA structure

Translation of the mRNA message into protein consists of initiation, elongation and termination steps. The substrate of translational control is not just naked mRNA, but mRNA covered with RNA-binding proteins (RBPs) forming ribonucleoprotein particles or RNPs. Often in combination with miRNAs, sequence-specific RBPs play important roles in translational regulation [51]. Many RBPs bind to structured elements, and binding is actually based on structural fidelity rather than primary sequence recognition [52,53]. Because of the altered tertiary structure (Fig 3, SF1) of the variant mRNA segment, the RBPs may not be able to bind properly to generate a fully functional RNP. Further, altered structure of the variant prevents proper pseudo-circularization needed for the action of 3′-UTR effectors on translation initiation at the 5′-end. These features have the potential to repress translation of select proteins in epilepsy patients. The altered mRNA segments bind quite strongly each additional miRNA (Table 4) leading to prediction of higher translational repression. It is reassuring that these inter-molecular hydrogen bonds in mRNA-miRNA complexes are consistent with the data in mirSNPDB.

### Febrile Seizures (FS) and MTLE

Temporal Lobe Epilepsy (TLE), is associated with focal seizures in the temporal lobe that houses the hippocampus and processes signals of memory, speech, vision and different types of emotions. TLE is the most common and difficult-to-treat type of epilepsy in adults [54]. Most cases of MTLE are sporadic and there are also suggestions of genetic contribution [55–59] although no genes have yet been strongly associated specifically with MTLE. Prolonged FS during childhood that potentially damage the hippocampus are often associated with a substantially elevated risk for future epilepsy, including TLE, after a gap of nearly 8–12 years [60,61]. This correlation is being researched further to identify possible genetic factors. A differentially expressed genes (DEG) study of MTLE with and without prolonged FS history has identified 496 genes as over-expressed in the former. The Protein-Protein Interaction (PPI) network analysis of these DEGs reveals that proteins *GRIN1*, *GRIN2A*, *SLC12A5* and *SLC1A2* form functionally crucial nodes in the most significant network-module [62]. In the present study on MTLE patients, we find SNVs affecting miRNA binding sites in the 3’-UTR regions of *GRIN2A*, *SLC1A3* and *SLC12A6*, genes identical or closely related to those occurring in the network-module (Table 2). Since miRNA binding in the 3’-UTR region is a mode of translation regulation, our observations are suggestive of the possible mechanism of linkage between FS and MTLE. Similarly, our finding of 3’-UTR SNVs in *HERC2* (Table 2) may be significant because altered expression of *HERC1*, which belongs to the same family as *HERC2*, is shown to be associated with FS [63].

## Materials and methods

### a) Genomics data

#### i) Exome sequences

RNA-seq data on 38 samples available as paired reads in the Short Read Archive (SRA) of NCBI were downloaded in the FASTQ format.

Thirty-five samples refer to MTLE patients, and three samples refer to the non-epileptic brain tissue treated as control in this RNA-seq analysis. The sequence of human genome hg-19 assembly available in “.fa” format was downloaded from the link featured in the UCSC Genome Browser.

#### ii) Epilepsy-related genes

The names of genes already known to be associated with epilepsy were assembled from the database CARPEDB (http://carpedb.ua.edu/) and the article by Wang et. at. [24]. We collected a total of 1073 genes as ‘already related to epilepsy’ genes.

### b) Software used

#### (i) RNA-Seq

The paired reads from the samples were used without further processing. They were mapped onto human genome assembly hg-19, using two software packages: Bowtie2 [64] and BWA [65]. The two sets of output Sequence Alignment Map (SAM) files were then converted into two sets of binary map files after sorting and indexing using SAMTOOLS software [66]. The variant calling on these two BAM files was by using the mpileup command of SAMTOOLS (version 0.1.19-96b5f2294a) [66] followed by BCFTOOLS (Version: 0.1.19-96b5f2294a) to generate the VCF file containing all information about the sequence variants. Only those variants which satisfied the two conditions of mapping quality > 30 and depth of read (DP) > 100 were retained for subsequent analysis. Analysis of these two sets of VCF files was done in two different ways. In the first method VCF files produced by BWE software were used, and variants present in all thirty-five drug-refractory MTLE patient samples but absent in the three-control genome were accepted as genuine variants. These variants were summed up using vcf-merge utility of VCFTOOLS (v0.1.11) [67] software. In the second method only the variants that are identified by both Bowtie2 and BWA-mem mappings were considered as genuine, irrespective of in how many samples a particular variant was observed. This final VCF style file was used for analysis in the variant effect predictor (ENSEMBL-VEP) web-Software from ENSMBL and has reported allele frequencies from the 1000 Genomes [68,69].

#### (ii) RNA structure

The RNA secondary structure was predicted using RNAFOLD server [70]. The secondary structure of mRNA-miRNA complex was predicted using two independent servers: IntaRNA [71] and Vienna RNAcofold [70]. The predicted structures were visualized using Forna [72] and RiboSketch [73].

#### (iii) Three-dimensional structure prediction, visualization and superposition

The three dimensional structure of the RNA which incorporates the input secondary structure was predicted Denovo using software Rosetta [74]. The software COOT [75], Pymol [76] were used for visualization and superposition of three dimensional structures.

#### (iv) MicroRNA identification for 3’UTR SNVs

We used two approaches to identify the miRNAs that would bind at the SNV site. Whatever miRNAs were common to both predictions were used for molecular modelling studies. In the first approach, the software RIblast [77] was used to identify from miRNA database, miRBase 22 [78], the miRNAs that would bind to the 11 residue query sequence with the variant base occupying position 6 in the query sequence. In the second approach, the miRNAs likely to bind at the SNV site were identified by searching the miRSNPDB database [79,80]. In the second approach, the miRNAs output as potential hits are predicted to be binding, by all three tools, mirTar [81], MIRANDA [82] and Targetscan [14].

## Supporting information

S1 Fig Secondary structure changes in GRIN2A, CACNB4, FBXO28, TDP2, ABHD12, RBPJ, PCMT1due to loss of miRNA binding sites. (TIF)

S2 Fig Three dimensional structures of the native/control as well as variant mRNA segments of additional genes. (TIF)

S3 Fig Hydrogen bonding interactions in the seed region of the modeled complex for genes that gained in miRNA binding sites. (TIF)

S1 Table RNA-seq data IDs from NCBI-SRA and quality of mapping to hg-19 genome. (DOCX)

S2 Table Epilepsy-associated genes carrying 3’-UTR SNVs and 5’-UTR SNVs. (DOCX)
