# Supplementary figures and images for: Identification of 3’-UTR single nucleotide variants and prediction of select protein imbalance in mesial temporal lobe epilepsy patients
Source: PLoS One. 2021 Jun 4;16(6):e0252475. doi: 10.1371/journal.pone.0252475 (PMC8177469; doi:10.1371/journal.pone.0252475)

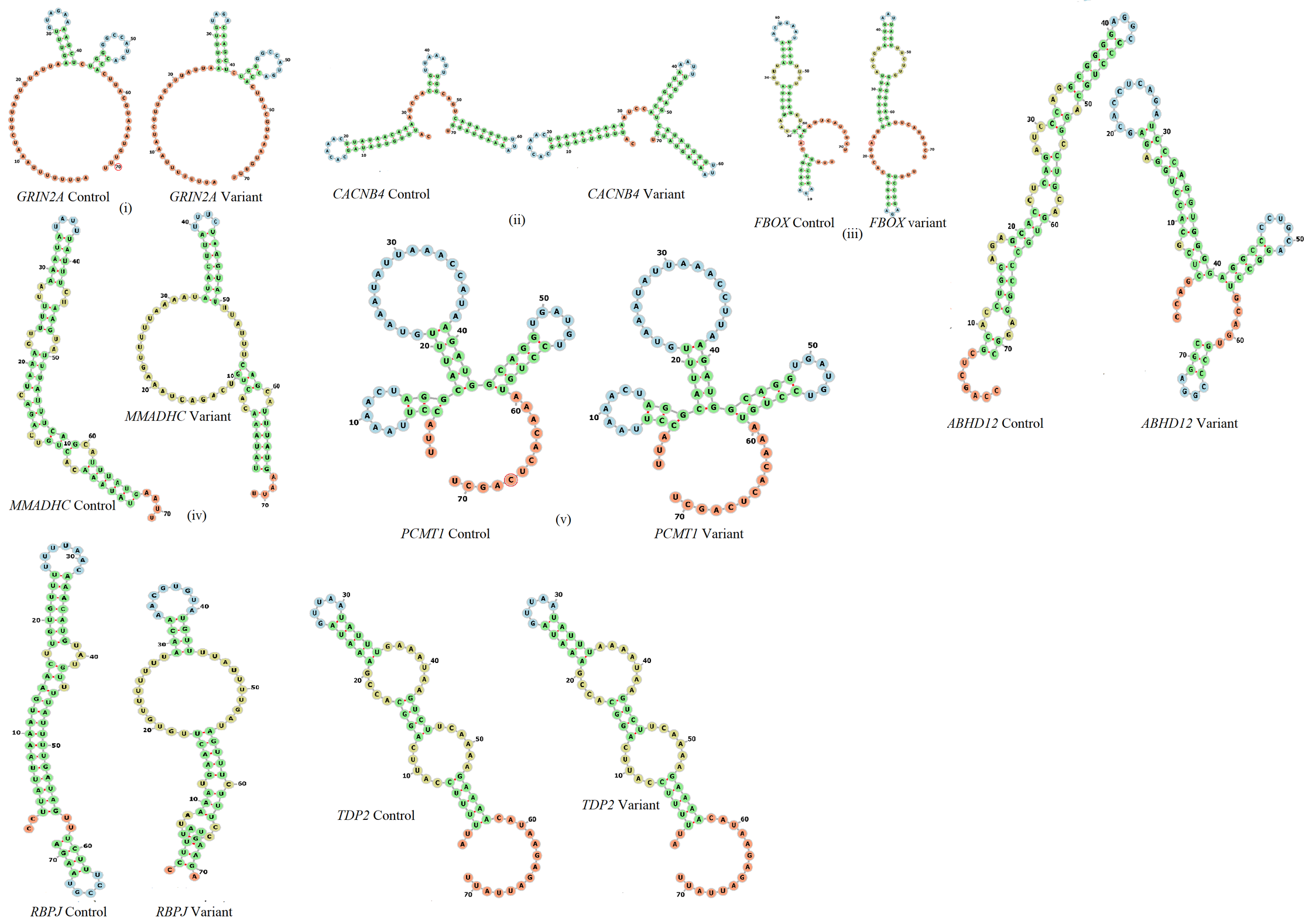

Supplement: S1 Fig — (TIF) [file pone.0252475.s001.tif]

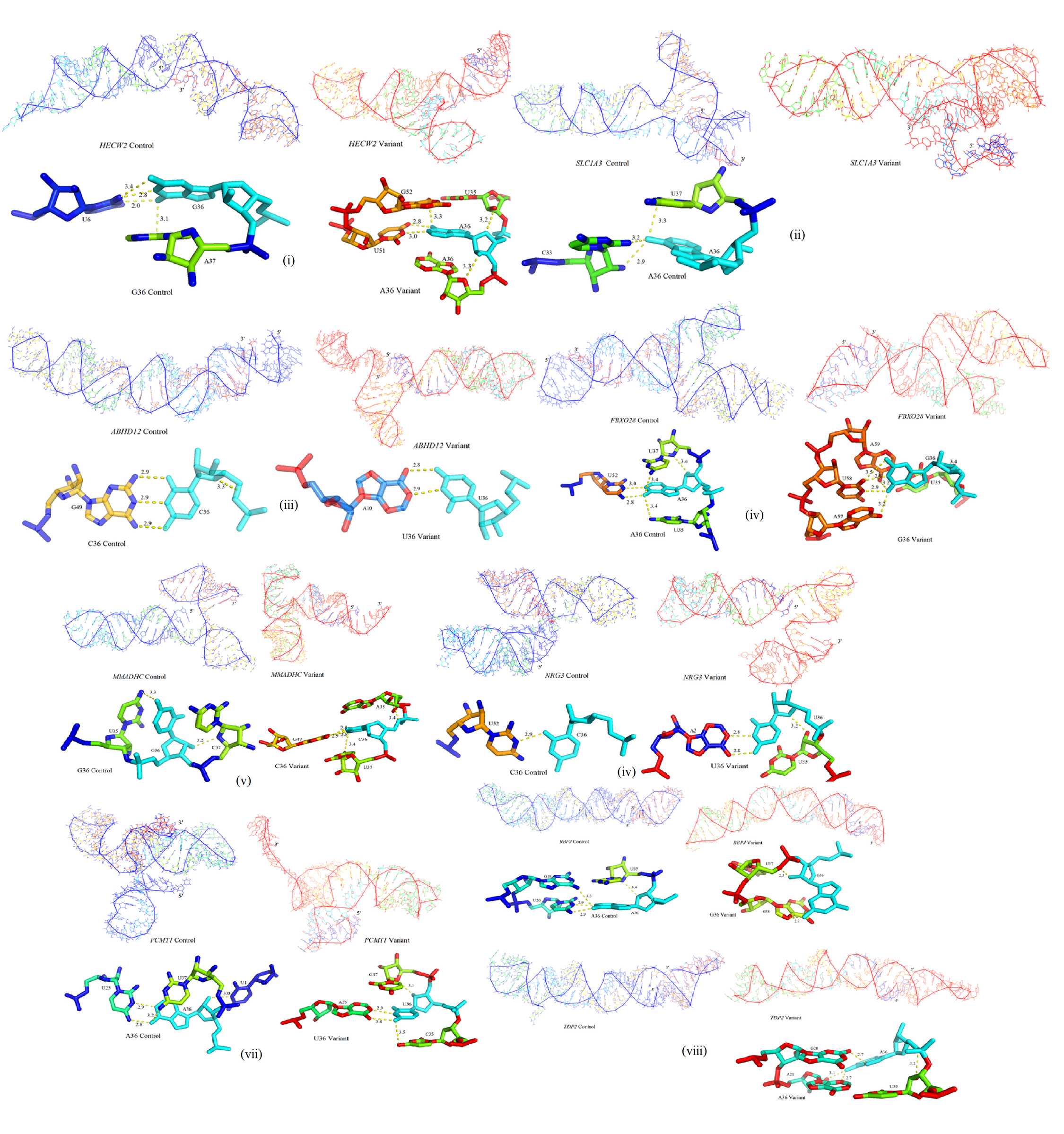

Supplement: S2 Fig — (TIF) [file pone.0252475.s002.tif]

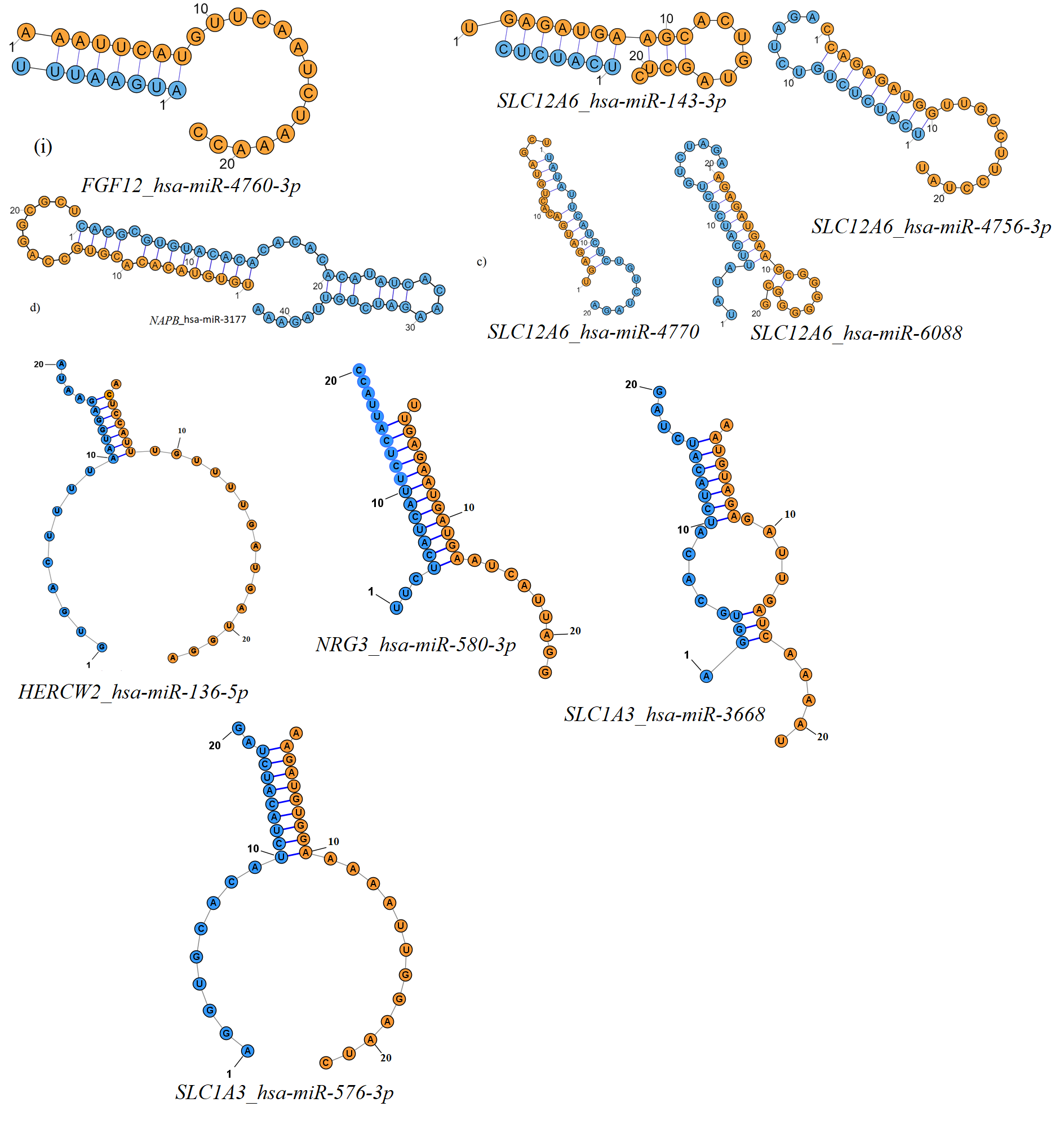

Supplement: S3 Fig — (TIF) [file pone.0252475.s003.tif]
