# Supplementary material for: Identification of 3’-UTR single nucleotide variants and prediction of select protein imbalance in mesial temporal lobe epilepsy patients
Source: PLoS One. 2021 Jun 4;16(6):e0252475. doi: 10.1371/journal.pone.0252475 (PMC8177469; doi:10.1371/journal.pone.0252475)
Supplement: S1 Table — (DOCX) [file pone.0252475.s004.docx]

Supplementary Table S1:

Table S1. RNA-seq data IDs from NCBI-SRA and quality of mapping to hg-19 genome.

| SRA_number | total_reads | mapped reads | unmapped_reads (multiply_mapped) | Reads above mapq>30 | Fraction overall_aligned | Average DP (depth of read) |
| --- | --- | --- | --- | --- | --- | --- |
| SRR1956809 | 21936455 | 21842669 | 93786 | 19511685 | 0.9957 | 11.483 |
| SRR1956833 | 65632276 | 54523199 | 11109077 | 43300084 | 0.8307 | 21.3603 |
| SRR2106309 | 130204546 | 129943300 | 261246 | 120194638 | 0.998 | 34.1615 |
| SRR2106310 | 130411147 | 129906722 | 504425 | 117970689 | 0.9961 | 29.1372 |
| SRR2106311 | 163045776 | 162484982 | 560794 | 151090925 | 0.9966 | 38.6168 |
| SRR2106312 | 141314874 | 140463271 | 851603 | 127276573 | 0.994 | 31.75 |
| SRR2106313 | 142096204 | 141838744 | 257460 | 132298131 | 0.9982 | 29.0755 |
| SRR2106314 | 138711915 | 137557654 | 1154261 | 125918053 | 0.9917 | 25.5609 |
| SRR2106315 | 163271227 | 162842655 | 428572 | 151822822 | 0.9974 | 34.5202 |
| SRR2106316 | 122328667 | 121719069 | 609598 | 111305618 | 0.995 | 24.8821 |
| SRR2106318 | 113112883 | 112868206 | 244677 | 104443364 | 0.9978 | 23.7846 |
| SRR2106320 | 117906941 | 116545123 | 1361818 | 105435628 | 0.9884 | 20.1829 |
| SRR2143509 | 136479117 | 135028470 | 1450647 | 121373502 | 0.9894 | 31.0802 |
| SRR2143510 | 125289412 | 124259795 | 1029617 | 107823388 | 0.9918 | 29.2061 |
| SRR2143511 | 116179946 | 115172760 | 1007186 | 105457838 | 0.9913 | 36.0932 |
| SRR2143512 | 130102671 | 119584770 | 10517901 | 93288496 | 0.9192 | 29.003 |
| SRR2143513 | 169779724 | 165843446 | 3936278 | 148839759 | 0.9768 | 104.109 |
| SRR2143514 | 171944307 | 166600015 | 5344292 | 138543701 | 0.9689 | 38.7372 |
| SRR9733948 | 85246798 | 85109011 | 137787 | 58230622 | 0.9984 | 9.7836 |
| SRR9733950 | 88751606 | 88591133 | 160473 | 59578567 | 0.9982 | 11.6581 |
| SRR9733952 | 70231572 | 70059773 | 171799 | 44643337 | 0.9976 | 7.75438 |
| SRR9733954 | 81922081 | 81785001 | 137080 | 60006855 | 0.9983 | 8.83583 |
| SRR9733956 | 85395456 | 85185777 | 209679 | 54118396 | 0.9975 | 8.34591 |
| SRR9733958 | 88080208 | 87941823 | 138385 | 61118798 | 0.9984 | 8.98511 |
| SRR9733960 | 91874279 | 91577263 | 297016 | 57987042 | 0.9968 | 8.41674 |
| SRR9733962 | 76158716 | 75914157 | 244559 | 55979976 | 0.9968 | 8.42061 |
| SRR9733964 | 87941615 | 87703593 | 238022 | 66582827 | 0.9973 | 11.1739 |
| SRR9733966 | 81425809 | 81142021 | 283788 | 61694983 | 0.9965 | 10.5962 |
| SRR9733968 | 88162357 | 87886933 | 275424 | 66086097 | 0.9969 | 10.9285 |
| SRR9733970 | 85699367 | 85003559 | 695808 | 63080352 | 0.9919 | 11.0392 |
| SRR9733972 | 84199981 | 84021636 | 178345 | 66067473 | 0.9979 | 17.9377 |
| SRR9733974 | 79433252 | 79178784 | 254468 | 61787758 | 0.9968 | 12.0378 |
| SRR9733976 | 87197800 | 86274123 | 923677 | 64774653 | 0.9894 | 11.3595 |
| SRR9733978 | 66163414 | 65541785 | 621629 | 49821904 | 0.9906 | 9.73046 |
| SRR9733980 | 63786402 | 63097873 | 688529 | 51234639 | 0.9892 | 13.7522 |
| SRR1957110 | 95813158 | 95583689 | 229469 | 73165907 | 0.9976 | 8.99624 |
| SRR9733981 | 79808813 | 79500474 | 308339 | 62282719 | 0.9961 | 12.0127 |
| SRR9733982 | 28855397 | 28793140 | 62257 | 25122896 | 0.9978 | 13.8856 |
