# Supplementary material for: Identification of 3’-UTR single nucleotide variants and prediction of select protein imbalance in mesial temporal lobe epilepsy patients
Source: PLoS One. 2021 Jun 4;16(6):e0252475. doi: 10.1371/journal.pone.0252475 (PMC8177469; doi:10.1371/journal.pone.0252475)
Supplement: S2 Table — (DOCX) [file pone.0252475.s005.docx]

Supplementary Table S2:

Table S2: Names of Epilepsy-associated genes carrying 3'-UTR SNVs and 5'-UTR SNVs.

| SNV type | Gene Names |
| --- | --- |
| 3'_UTR: | *AARS, AASS, ABAT, ABCB1, ABCC1, ABHD12, ACADSB, ACOT7, ACOX1, ACTB, ACTG1, ACVR1, ADAM22, ADD3, ADK, ADNP, AGL, AGPS, AHI1, AIMP1, AKT2, AKT3, ALDH4A1, ALDH5A1, ALDH7A1, ALG1, ALG11, ALG2, AMACR, AMPD2, ANK3, ANKH, AP1S2, AP3D1, APP, AQP4, ARFGEF2, ARHGDIA, ARHGEF9, ARID1A, ARID1B, ARNT2, ASAH1, ATP13A2, ATP1A2, ATP5A1, ATP6AP1, ATP6AP2, ATP6V0A2, ATP6V0C, ATP8A2, ATXN10, AUTS2, BCAP31, BCL10, BDNF, BMP4, BRAF, BSN, C18orf25, CACNA1A, CACNA1D, CACNA1E, CACNA2D1, CACNB4, CACNG2, CACNG3, CALN1, CAMTA1, CBL, CC2D2A, CCDC88A, CCM2, CD46, CD59, CDK5, CDKL5, CELF4, CEP290, CERS1, CHD1L, CHD2, CHD3, CHL1, CHN1, CHRNB2, CLCN4, CLCN6, CLIC2, CLN5, CLN8, CLPB, CLPP, CLSTN1, CNNM2, CNR1, CNTN2, CNTNAP2, COG6, COG7, COL4A3BP, COL6A3, COQ4, COQ9, COX10, CPT2, CREBBP, CRYAB, CSF1R, CSMD3, CSNK1G1, CSTB, CTNND2, CTSD, CTSF, CUX1, CYB5R3, DAPK1, DEAF1, DGKD, DHCR24, DIAPH1, DIP2C, DKC1, DLD, DLG2, DLG3, DMD, DNAJC5, DNAJC6, DNM1L, DNM3, DOCK7, DOCK8, DPAGT1, DPYD, DPYSL2, DYRK1A, EARS2, EIF2B1, EIF2S1, ELMO1, ELOVL4, EPG5, EPHA5, EPM2A, ERBB4, ERCC6, EXOC6B, EXT2, FAR1, FASTKD2, FAT4, FBXO28, FGF12, FGF2, FGFR2, FGFR3, FKTN, FLNA, FMN2, FMR1, FOSB, FRRS1L, FTO, GABBR2, GABRA1, GABRB1, GABRB2, GABRD, GABRG2, GAL, GALC, GATAD2B, GBE1, GCH1, GDI1, GFAP, GFM1, GIPC1, GJC2, GJD2, GK, GLUL, GMPPB, GNA11, GNAO1, GNAQ, GNB1, GOSR2, GPHN, GPR56, GPSM2, GPX4, GRIA3, GRIK2, GRIN2A, GRIN2B, GRM1, GUCY1A3, GUF1, HACE1, HADHA, HCN1, HCN2, HECW2, HEG1, HEPACAM, HERC2, HGSNAT, HIBCH, HIP1, HIVEP2, HNRNPH1, HNRNPU, HS2ST1, HSD17B4, HTR2A, HTT, HUWE1, IDS, IER3IP1, IFNAR2, IGSF8, INPP4A, INSR, ITGB1BP1, ITPR1, JAM3, JRK, KARS, KAT6A, KATNB1, KCNA1, KCNA2, KCNAB1, KCNAB2, KCNB1, KCNC1, KCND2, KCND3, KCNH1, KCNJ10, KCNJ2, KCNJ6, KCNMA1, KCNMB3, KCNN3, KCNQ2, KCNQ3, KCNV1, KCTD7, KDM6A, KIAA1456, KIF1A, KIF3C, KIF5C, KLF13, KMT2A, KPTN, KRAS, KRIT1, L1CAM, LAMB1, LARS2, LGI3, LGI4, LIAS, LPHN2, LRPPRC, LRRK2, MAF, MAGEL2, MAGI2, MAN1B1, MAN2A2, MANBA, MAOA, MAPK10, MAPRE2, MBD5, MBTPS2, MCCC2, MCM9, ME2, MED13L, MFSD8, MID2, MLC1, MLLT3, MMADHC, MOCS2, MRI1, MRPS22, MSC, MTFMT, MTHFR, MTO1, MTOR, MTR, MYO9B, NADK2, NALCN, NAPB, NARS2, NAT8L, NDP, NDUFAF6, NDUFS4, NDUFS8, NEDD4L, NF1, NHLRC1, NIN, NIPA1, NIPA2, NKAIN3, NLGN1, NONO, NRG3, NRXN1, NSD1, NUBPL, OPA1, OPHN1, PAK3, PARK2, PCDH15, PCDH19, PCDH7, PCDHB4, PCMT1, PDE10A, PDHA1, PDHX, PDSS2, PDYN, PEX13, PEX19, PEX3, PEX5, PEX7, PGAP1, PGAP2, PGK1, PHF6, PIGA, PIGM, PIGN, PIGV, PIK3AP1, PLCB1, PLD1, PLXNB2, PMP22, PNPLA8, PNPO, PNPT1, PODXL, POLG, POLR3B, POMT1, PPP1R3C, PPP2R1A, PPT1, PRDM8, PRICKLE1, PRICKLE2, PRNP, PRODH, PROS1, PRRC2B, PRRT2, PSAT1, PSEN1, PSEN2, PSPH, PTEN, PTGS2, PURA, QDPR, RAB18, RAB39B, RANBP2, RAPGEF6, RBFOX1, RBFOX3, RBPJ, RELN, RFX3, RMND1, RNASET2, ROGDI, RRM2B, RYR3, S100B, SACS, SCARB2, SCN1B, SCN2A, SCN3A, SCN8A, SDHD, SEMA5B, SEPSECS, SETD2, SETD5, SGCE, SLC12A5, SLC12A6, SLC16A1, SLC17A5, SLC1A1, SLC1A2, SLC1A3, SLC1A4, SLC25A22, SLC35A3, SLC39A8, SLC46A1, SLC4A3, SLC6A1, SLC6A12, SLC6A8, SLC7A11, SLC8A1, SLC9A6, SLC9A9, SMARCA2, SMARCB1, SMARCE1, SNAP25, SNIP1, SOX2, SOX5, SPTAN1, SPTLC2, SQSTM1, SRGAP2, ST3GAL3, ST5, STAT1, STRADA, STT3A, STT3B, STX1B, STXBP1, SUCLA2, SV2A, SYN2, SYNJ1, SYP, SYT14, SZT2, TANGO2, TBC1D24, TBL1XR1, TBP, TCF4, TDP2, TECPR2, TENM2, THAP1, THRB, TK2, TMEM70, TNK2, TOR1A, TPP1, TRAPPC11, TSC1, TSEN15, TSPYL4, TUBA1A, TUBB4A, TXN2, UBA5, UBE3A, UBR5, UNC80, UQCC2, USP9X, VPS35, VPS53, WDR19, WWOX, XK, YWHAE, ZBTB18, ZEB2, ZFYVE20, ZFYVE26, ZMYND11, ZMYND8, ZNF12, ZNF182, ZNF354A, ZSWIM6* |
| 5'_UTR: | *ABAT, ANK3, ATXN10, BMP4, C12orf57, CACNA2D1, CALM2, CELF4, CEP290, CLSTN1, CSF1R, CTNND2, DPYSL2, GABRG2, GFAP, GNAQ, GNPAT, GRIA3, GUCY1A3, HEPACAM, HNRNPH1, KAT6A, KCNAB1, KCNQ2, KIF5C, LRFN5, LRPPRC, MYT1L, NLGN1, NRXN1, PEX19, PPP2R1A, PRODH, PTEN, QDPR, RNASET2, RTN4IP1, SACS, SLC7A11, SLC9A9, ST7, STK11, SV2A, TRIM8, UBE2A,* |
